# Longitudinal changes in forced expiratory volume in 1 s in patients with eosinophilic chronic obstructive pulmonary disease

**DOI:** 10.1186/s12890-022-01873-8

**Published:** 2022-03-16

**Authors:** Yong Suk Jo, Ji-Yong Moon, Yong Bum Park, Yee Hyung Kim, Soo-Jung Um, Woo Jin Kim, Hyoung Kyu Yoon, Kwang Ha Yoo, Ki-Suck Jung, Chin Kook Rhee

**Affiliations:** 1 Division of Pulmonary and Critical Care Medicine, Department of Internal Medicine, Seoul St. Mary’s Hospital, College of Medicine, The Catholic University of Korea, 222 Banpo-daero, Seocho-gu, Seoul, 06591 South Korea; 2 Department of Internal Medicine, Hanyang University College of Medicine, Seoul, South Korea; 3 Division of Pulmonary, Allergy, and Critical Care Medicine, Department of Internal Medicine, Hallym University Kangdong Sacred Heart Hospital, Seoul, South Korea; 4 Department of Pulmonary, Allergy and Critical Care Medicine, Kyung Hee University Hospital at Gangdong, Kyung Hee University School of Medicine, Seoul, South Korea; 5 Division of Respiratory Medicine, Department of Internal Medicine, Dong-A University College of Medicine, Dong-A University Medical Center, Busan, South Korea; 6 Department of Internal Medicine and Environmental Health Center, Kangwon National University Hospital, Chuncheon, South Korea; 7 Division of Pulmonary and Critical Care Medicine, Department of Internal Medicine, Yeouido St. Mary’s Hospital, College of Medicine, The Catholic University of Korea, Seoul, South Korea; 8 Division of Pulmonary, Allergy and Critical Care Medicine, Department of Internal Medicine, Konkuk University School of Medicine, Seoul, South Korea; 9 Department of Pulmonary, Allergy and Critical Care Medicine, Hallym University Sacred Heart Hospital, Anyang, South Korea

**Keywords:** COPD, Blood eosinophil count, Exacerbation, Forced expiratory volume in 1 s, Longitudinal change

## Abstract

**Background:**

Data on changes in lung function in eosinophilic chronic obstructive pulmonary disease (COPD) are limited. We investigated the longitudinal changes in forced expiratory volume in 1 s (FEV_1_) and effects of inhaled corticosteroid (ICS) in Korean COPD patients.

**Methods:**

Stable COPD patients in the Korean COPD subgroup study (KOCOSS) cohort, aged 40 years or older, were included and classified as eosinophilic and non-eosinophilic COPD based on blood counts of eosinophils (greater or lesser than 300 cells/μL). FEV_1_ changes were analyzed over a 3-year follow-up period.

**Results:**

Of 627 patients who underwent spirometry at least twice during the follow up, 150 and 477 patients were classified as eosinophilic and non-eosinophilic, respectively. ICS-containing inhalers were prescribed to 40% of the patients in each group. Exacerbations were more frequent in the eosinophilic group (adjusted odds ratio: 1.49; 95% confidence interval: 1.10–2.03). An accelerated FEV_1_ decline was observed in the non-eosinophilic group (adjusted annual rate of FEV_1_ change: − 12.2 mL/y and − 19.4 mL/y for eosinophilic and non-eosinophilic groups, respectively). In eosinophilic COPD, the adjusted rate of annual FEV_1_ decline was not significant regardless of ICS therapy, but the decline rate was greater in ICS users (− 19.2 mL/y and − 4.5 mL/y, with and without ICS therapy, respectively).

**Conclusions:**

The annual rate of decline in FEV_1_ was favorable in eosinophilic COPD compared to non-eosinophilic COPD, and ICS therapy had no beneficial effects on changes in FEV_1_.

**Supplementary Information:**

The online version contains supplementary material available at 10.1186/s12890-022-01873-8.

## Background

Chronic obstructive pulmonary disease (COPD) is a progressive inflammatory airway disease characterized by reduced airflow, and is a leading cause of mortality worldwide [1].

COPD is regarded as heterogeneous condition and chronic airway inflammation in COPD is thought to be driven by neutrophils and lymphocytes [2]. This type of airway inflammation is a prominent feature in most COPD patients unlike in asthmatic patients, and related to the degree of airflow limitation [3].

Airway inflammation in asthma is mediated by type-2 T helper (Th2) cells and eosinophils, associated with an exacerbation risk and poor disease control following inhaled corticosteroid (ICS) withdrawal [4, 5]. The presence of eosinophils has been reported in the blood or sputum of 30–40% COPD patients [6]. Excluding patients with clinical features suggestive of asthma, such as bronchial hyperresponsiveness, history of asthma, atopy, and reversible airflow limitation, a subset of COPD patients demonstrates eosinophilic airway inflammation and responds to corticosteroid therapy [7–9].

Several studies have reported a greater risk for COPD exacerbation with higher blood counts of eosinophils, although the relationship remains controversial. In the general population, spirometry-based COPD patients experience more exacerbations with high blood eosinophil counts [10, 11]. Although higher eosinophil levels increase the risk for COPD exacerbation, they are also associated with a greater risk reduction after the use of ICS [12–14].

However, few studies have investigated the effects of blood levels of eosinophils on lung function. In this study, we evaluated longitudinal changes in forced expiratory volume in 1 s (FEV_1_) in patients with eosinophilic COPD, and analyzed the effects of ICS on this parameter.

## Methods

### Study population

Data were obtained from the Korean COPD Subgroup Study (KOCOSS; 2012–2019), an ongoing prospective multicenter observational cohort, which has recruited COPD patients from 48 referral hospitals in the Republic of Korea [15]. The inclusion criteria were (1) age ≥ 40 years; and (2) COPD diagnosed by pulmonologists based on respiratory symptoms and spirometry-confirmed fixed airflow limitation (post-bronchodilator FEV_1_/forced vital capacity (FVC) < 0.70). Only patients with blood eosinophil count data at baseline enrollment during stable state and followed up at least 3 years from enrollment were included in the analyses.

### Clinical data

KOCOSS cohort contained detailed information regarding sociodemographics (including age, sex, smoking history, and education level), symptoms (chronic cough or persistent phlegm ˃ 3 months), dyspnea severity based on the Modified Medical Research Council Dyspnea Scale (mMRC), and quality of life based on the COPD assessment test (CAT) and the St George’s Respiratory Questionnaire (SGRQ). Data on comorbidities, including cardiovascular disease, tuberculosis, and asthma, were also collected. The treatment of the subjects depended on their attending pulmonologist.

The KOCOSS cohort patients were followed-up at least every 6 months, and moderate and severe exacerbations were recorded at each visit. Moderate exacerbation was defined as an exacerbation leading to an outpatient-clinic visit earlier than scheduled and prescribed systemic steroids and/or antibiotics, whereas severe exacerbation was defined as leading to an emergency department visit or hospitalization.

### Pulmonary function test

Spirometry was performed based on American Thoracic Society and European Respiratory Society guidelines [16]. Absolute FEV_1_ values were obtained, and the predicted percentage values (% pred) for FEV_1_ were calculated using an equation developed for the Korean population [17]. Positive bronchodilator response (BDR) was defined as post-bronchodilator FEV_1_ increase in 12% or more, and 200 mL from baseline. Subjects were followed-up at least every 6 months, and spirometry was performed annually.

### Statistical analysis

Baseline characteristics were expressed as means ± standard deviations and absolute numbers with percentages. Continuous and categorical variables were compared using t-test and chi-square test, respectively.

In this study, we assess the longitudinal changes in post-bronchodilator FEV_1_ (mL). To exclude the immediate effects of bronchodilator treatment, lung function data used for longitudinal analysis were collected one year after enrollment.

Although variability on serial blood eosinophil counts may be concernable, over 300 cells/µL is regarded as threshold for predicting increased risk of exacerbation and high likelihood of benefit with ICS [18, 19]. We compared the longitudinal FEV_1_ change between eosinophilic (blood eosinophil count ≥ 300 cells/μL) and non-eosinophilic (blood eosinophil count < 300 cells/μL) COPD, using a linear mixed-effects model with random intercepts and slopes. We analyzed mean annual rates of FEV_1_ change based on multiple covariates: age, sex, smoking (pack-years), and body mass index (BMI). Then, we analyzed FEV_1_ changes based on ICS usage.

All tests were two-sided and a p-value less than.05 was considered statistically significant. Statistical analyses were performed using Stata (v. 16; StataCorp, College Station, TX, USA).

## Results

### Study subjects

**Fig. 1:**
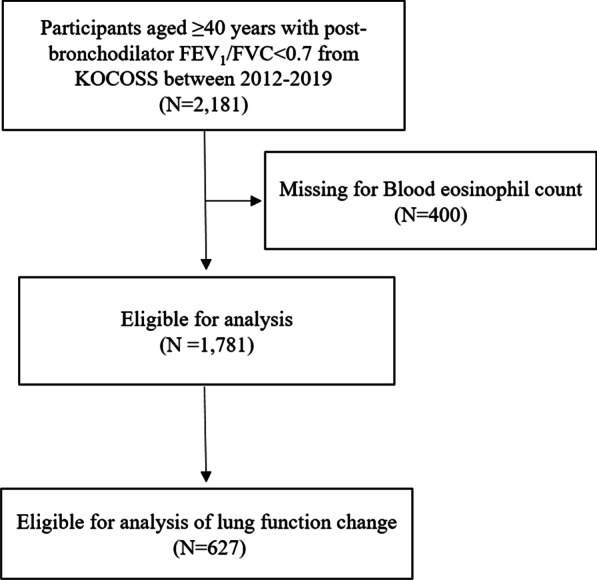
Study flow. COPD, chronic obstructive pulmonary disease; KOCOSS, Korean COPD subgroup study; FEV_1_, forced expiratory volume in 1 s; FVC, forced vital capacity;

A total of 2,181 COPD patients were enrolled between January 2012 and December 2019. FEV_1_ change analysis based on blood counts of eosinophils was available for 627 patients (Fig. 1). Comparison of clinical features between eosinophilic and non-eosinophilic COPD is shown in Table 1. Eosinophilic COPD was more prevalent in males (98.7% and 91.2%, for eosinophilic and non-eosinophilic groups, respectively). There were no significant differences in age, smoking status, symptoms, and quality of life. Past exacerbations were more common in eosinophilic COPD group, compared to non-eaosinophilic group (25.3% and 18.5%, respectively), but the difference was not statistically significant.

**Table 1:** Baseline characteristics of eosinophilic and non-eosinophilic COPD

Characteristics (n = 627)	Non-eosinophilic COPD (n = 477)	Eosinophilic COPD (n = 150)	P value
Age (years)	68.8 ± 7.5	67.8 ± 7.4	0.150
Sex (male)	435 (91.2)	148 (98.7)	0.002
Former smoker	437 (91.6)	139 (92.7)	0.253
Smoking pack-year	38.5 ± 25.4	43.1 ± 26.7	0.060
BMI (kg/m^2^)	23.1 ± 3.3	23.1 ± 3.3	0.894
Education (above high school)	55 (11.5)	29 (19.3)	0.045
Symptoms			
Cough > 3 months (n = 218)	80 (16.9)	20 (13.0)	0.078
Phlegm > 3 months (n = 240)	91 (19.2)	30 (19.5)	0.083
mMRC	1.35 ± 0.84	1.31 ± 0.83	0.617
Quality of life			
CAT score	14.8 ± 7.7	15.1 ± 7.9	0.590
SGRQ score			
Symptoms	41.7 ± 20.4	44.7 ± 22.8	0.122
Impacts	23.9 ± 22.6	26.6 ± 22.3	0.207
Activity	44.1 ± 26.1	48.3 ± 27.2	0.087
Total	33.1 ± 20.3	36.3 ± 20.9	0.094
Past exacerbation (n = 622)	88 (18.5)	38 (25.3)	0.093
Past severe exacerbation	39 (8.2)	11 (7.3)	0.423

The unit of eosinophil count is cell/μL

BMI, body mass index; CAT, Chronic Obstructive Pulmonary Disease Assessment Test; COPD, chronic obstructive pulmonary disease; mMRC, modified Medical Research Council; SGRQ, St. George's Respiratory Disease Questionnaire

Baseline pulmonary function test results are shown in Table 2. More than 60% of the patients in both groups were GOLD stage 1 or 2 (FEV_1_ ≥ 50% of predicted value), and there were no significant differences in the severity of airflow limitation and exercise capacity. Mean blood counts of eosinophils were higher in the eosinophilic COPD group (546.1 vs. 141.8 cells/μL).

**Table 2:** Lung function parameters and inhaler prescription status

Spirometry	Non-eosinophilic COPD (n = 477)	Eosinophilic COPD (n = 150)	P value
Post-BD FEV_1_, L	1.68 ± 0.58	1.65 ± 0.52	0.606
Post-BD FEV_1_, %predicted	58.3 ± 18.2	55.8 ± 16.8	0.136
Post-BD FVC, L	3.29 ± 0.80	3.22 ± 0.73	0.305
Post-BD FVC, %predicted	80.9 ± 15.7	77.2 ± 15.8	0.014
Post-BD FEV_1_/FVC	51.0 ± 11.9	51.6 ± 11.7	0.568
DLCO, % (n = 553)	64.7 ± 18.4	63.7 ± 19.4	0.591
BDR positivity	54 (11.3)	23 (15.3)	0.192
GOLD stage			
1 (FEV_1_ ≥ 80)	59 (12.4)	14 (9.3)	
2 (50% ≤ FEV_1_ < 80%)	259 (54.3)	81 (54.0)	0.591
3 (30% ≤ FEV_1_ < 50%)	137 (28.7)	45 (30.0)	
4 (FEV_1_ < 30%)	22 (4.6)	10 (6.7)	
Exercise capacity, 6MWD (m) (n = 523)	399.6 ± 117.3	392.6 ± 95.7	0.540
Blood eosinophil count (cell/μL)	141.8 ± 71.8	546.1 ± 404.4	< 0.001
Medication			
ICS	3 (0.6)	0	
Mono bronchodilator	129 (27.0)	51 (34.0)	
Dual bronchodilator	70 (14.7)	15 (10.0)	0.220
ICS/LABA	63 (13.2)	14 (9.3)	
Triple	130 (27.3)	47 (31.3)	
Any ICS	196 (41.1)	61 (40.7)	0.827

Unit of eosinophil count is cell/μL

BDR, bronchodilator response; DLCO, diffusion capacity of carbon monoxide; FEV1, forced expiratory volume in 1 s; FVC, forced vital capacity; GOLD, Global Initiative for Chronic Obstructive Lung Disease; ICS, inhaled corticosteroid; LABA, long acting beta2 receptor agonist; LAMA, long acting muscarinic receptor agonist; PDE4 inhibitor, phosphodiesterase-4 inhibitor; 6MWD, 6-min walk distance

ICS therapy, either alone or in combination with long-acting β2-agonists (LABA) and/or long-acting muscarinic antagonists (LAMA), was prescribed in 41.1% and 40.7% in non-eosinophilic and eosinophilic groups, respectively.

### Exacerbation risk

Exacerbations were identified in 414 of the 627 patients. Moderate to severe exacerbations occurred in 71.0% of the patients (294/414), and 20.8% of the patients (86/414) experienced frequent exacerbations (≥ 2 events/year).

Exacerbations were occurred in 76.6% (82/107) of eosinophilic COPD group, while in 69.1% (212/307) of non-eosinophilic group. The exacerbation risk was significantly higher in the eosinophilic group compared to the non-eosinophilic group (adjusted odds ratio [aOR]: 1.49; 95% confidence interval [CI]: 1.10–2.03; p < 0.05). However, the statistical significance was lost with adding ICS therapy as covariate (aOR: 1.63; 95% CI: 0.89–2.99) (Table 3).

**Table 3:** The risk of exacerbation in patients with eosinophilic COPD compared with non-eosinophilic COPD

	Prevalence of AE^†^	OR (95% CI)	Adjusted OR (95% CI)		
			Model 1	Model 2	Model 3
Moderate to severe AE	82/107 (76.6%) vs 212/307 (69.1%)	1.47 (1.10–1.97)*	1.54 (1.14–2.09)*	1.49 (1.10–2.03)*	1.63 (0.89–2.99)
Frequent AE (≥ 2/year, n = 414)	26/107 (24.3%) vs 60/307 (19.5%)	1.32 (0.98–1.79)	1.39 (1.01–1.91)*	1.39 (0.99–1.93)	1.32 (0.71–2.45)

AE, acute exacerbation; BMI, body mass index; CI, confidence interval; COPD, chronic obstructive pulmonary disease; ICS, inhaled corticosteroid; OR, odds ratio; PY, pack-year

Model 1 adjusted for age, sex, BMI and PY

Model 2 adjusted for age, sex, BMI, PY, and past AE

Model 2 adjusted for age, sex, BMI, PY, past AE and ICS use

*p < 0.05

^†^Prevalence of AE of eosinophilic versus non-eosinophilic COPD patients was presented

### *Longitudinal FEV*_*1*_* change*

**Fig. 2:**
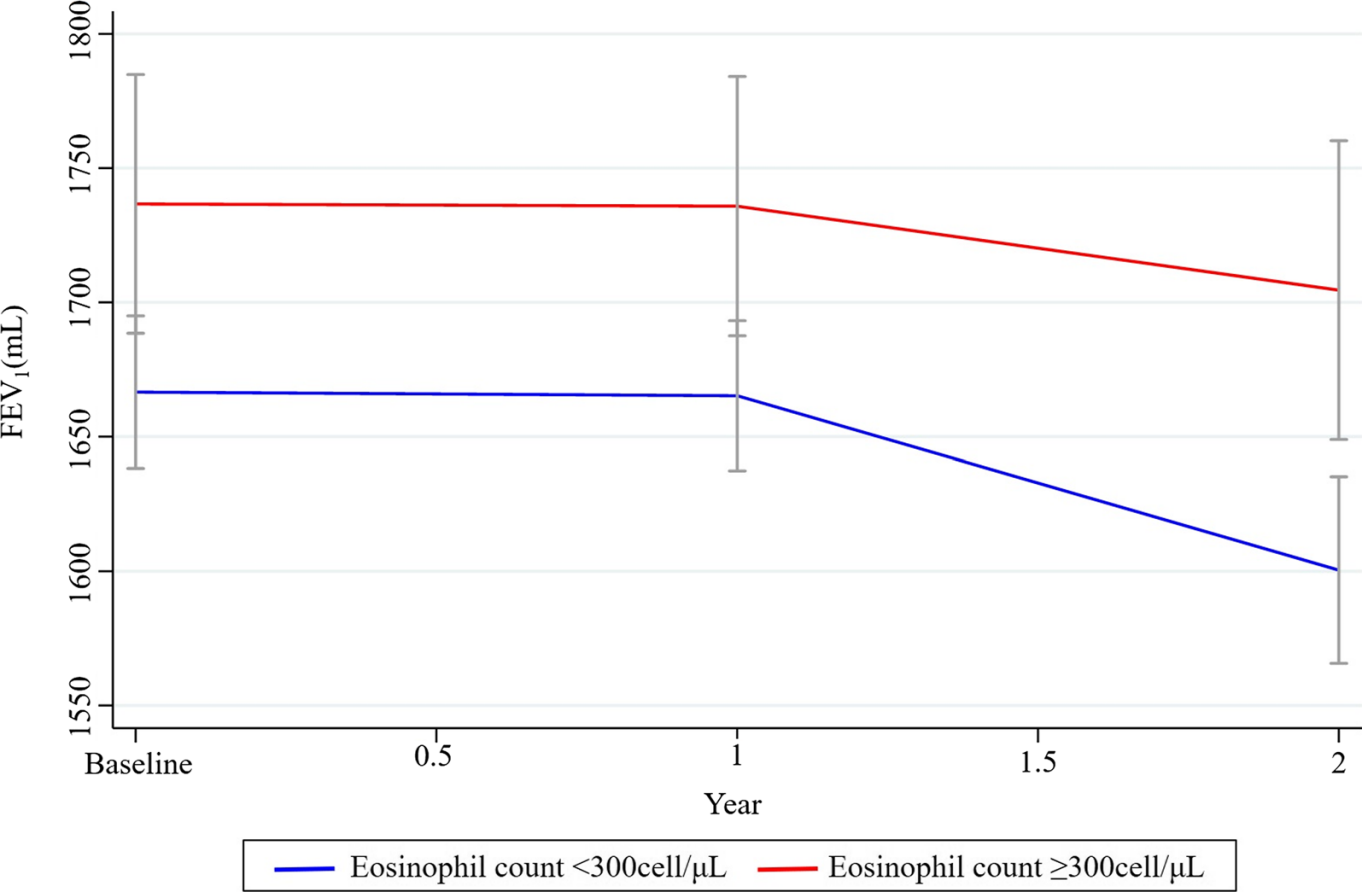
Changes of FEV_1_ over follow-up period in patients with eosinophilic and non-eosinophilic COPD. COPD, chronic obstructive pulmonary disease; FEV_1_, forced expiratory volume in 1 s

A greater FEV_1_ decline rate was observed in the non-eosinophilic group, compared to the eosinophilic group (− 19.6 mL/y and − 12.9 mL/y, respectively) (Fig. 2). The adjusted annual FEV_1_ declined significantly in the non-eosinophilic COPD group (− 19.4 mL/y). Although the adjusted annual FEV_1_ also decreased in the eosinophilic COPD group, the rate of decline was not significant (− 12.2 mL/y) (Table 4). In the eosinophilic COPD group, the adjusted annual FEV_1_ decline rate was not significant regardless of ICS therapy, but the decline rate was greater in ICS users (− 19.2 mL/y and − 4.5 mL/y, with and without ICS therapy, respectively) (Table 5). The exact values of FEV_1_ changes during two years of follow-up between eosinophilic and non-eosinophilic COPD are shown in Additional file 1: Figure S1.

**Table 4:** Annual FEV_1_ decline rate in eosinophilic and non-eosinophilic COPD

Non-eosinophilic COPD (n = 477)			Eosinophilic COPD (n = 150)		
Adjusted annual rate of change in FEV_1_		P value	Adjusted annual rate of change in FEV_1_		P value
Crude	− 19.6 ± 6.1	0.001	Crude	− 12.9 ± 10.1	0.204
Adjusted*	− 19.4 ± 6.2	0.002	Adjusted^*^	− 12.2 ± 10.3	0.233

BMI, body mass index; COPD, chronic obstructive pulmonary disease; FEV_1_, forced expiratory volume in 1 s; PY, pack-year

Data are shown as mean ± standard error

*Mixed-effect linear regression analysis was performed, which included the following covariates: age, sex, BMI and smoking PY

**Table 5:** Annual FEV_1_ decline rate in eosinophilic COPD according to the use of ICS

Without any ICS (n = 66)			With any ICS (n = 61)		
Adjusted annual rate of change in FEV_1_		P value	Adjusted annual rate of change in FEV_1_		P value
Crude	− 4.3 ± 18.3	0.815	Crude	− 20.7 ± 18.4	0.260
Adjusted*	− 4.5 ± 18.5	0.807	Adjusted^*^	− 19.2 ± 18.7	0.305

BMI, body mass index; COPD, chronic obstructive pulmonary disease; FEV_1_, forced expiratory volume in 1 s; ICS, inhaled corticosteroid; PY, pack-year

Data are shown as mean ± standard error

*Mixed-effect linear regression analysis was performed, which included the following covariates: age, sex, BMI and smoking PY

## Discussion

There is limited data regarding the effects of ICS on FEV_1_ change in COPD. We hypothesized that the rate of FEV_1_ change differed based on the baseline blood eosinophil counts and ICS therapy. The adjusted annual rate of FEV_1_ change declined significantly in the low-blood-eosinophil group, compared to the high-blood-eosinophil group, although the difference was not statistically significant. Analyses of ICS effects on FEV_1_ change in high-blood eosinophil group showed no statistically significant differences.

It is widely accepted that COPD develops because of a genetic susceptibility for progressive airway inflammation, triggered by complex environmental factors over time, such as smoking, pollutants, and allergens, which leads to irreversible damage and airflow obstruction [3, 20]. Typically, airways of COPD patients exhibit increased CD8 + T cells and neutrophils. Although Th2 inflammatory pathway is considered a feature of asthma rather than COPD, 30–40% of COPD patients demonstrated eosinophilic-inflammation [6]. These heterogeneity of disease are now accepted as various clinical phenotypes with different pathophysiologies and endotypes. Therefore, response to therapies might also be variable. The ECLIPSE (Evaluation of COPD Longitudinally to Identify Predictive Surrogate End points) cohort study reported that 37.4% of COPD patients had persistently high (≥ 2%), 13.6% had persistently low (< 2%), and 49% had variable eosinophil counts over a 3-year follow-up period [6]. A subset of COPD patients demonstrate eosinophilic inflammation either in stable period or during acute exacerbations. Blood eosinophilia was associated with increased exacerbation risk, and this phenotype could benefit from corticosteroid therapy directed against eosinophilic inflammation [13, 14, 21–23]. A recent trial targeted eosinophilic inflammation (IL-5) to reduce exacerbation risk in eosinophilic COPD patients, suggesting that selectively eosinophilic-pathway blockers may be effective for such patients [24]. Elevated peripheral blood eosinophil counts in COPD may be used to identify patients who are expected to have favorable response to ICS therapy or biologics targeting Th2 inflammatory pathway. Conversely, ICS withdrawal in eosinophilic COPD leads to increases exacerbation risk [25]. Following ICS withdrawal, trough FEV_1_ decreased and exacerbation risk increased only in the high eosinophil group [26].

Most trials assess the impact of ICS on COPD using exacerbation risk as the outcome, therefore data regarding ICS effects on FEV_1_ change is limited. A post hoc study reported favorable effects of extrafine beclomethasone dipropionate and formoterol fumarate, compared to formoterol fumarate alone, on FEV_1_ change in the highest blood eosinophil quartile (≥ 279.8 cells/μL). In this trial, severe COPD patients with FEV_1_ of 30–50% of predicted value, and at least one exacerbation in the previous year were included [27].

A study on effects of ICS monotherapy on disease progression in moderate to severe COPD reported no differences in the rate of decline of post-bronchodilator FEV_1_ between fluticasone propionate and placebo over 3 years. However, when analyzed using baseline blood eosinophil levels, the rate of FEV_1_ decline was significantly lower in the fluticasone propionate group compared to placebo in the ≥ 2% eosinophil group (− 40.6 mL/y and − 74.5 mL/y, respectively; p = 0.003)[22].

In the present study, annual rate of FEV_1_ change declined significantly in the lower blood eosinophil group. Analyses of ICS effects on longitudinal FEV_1_ change in high-blood-eosinophil group revealed a greater decline rate in ICS users. Although we demonstrated increased exacerbation risk in the eosinophilic COPD group, consistent with previous studies [10–12], the annual FEV_1_ decline rate was low in eosinophilic COPD compared to non-eosinophilic COPD, and ICS provided no benefits. Unlike previous trials [22, 27], only 20% of subjects had previous exacerbations and approximately 66% had mild to moderate FEV_1_ severities in the KOCOSS cohort. Different airflow obstruction severities and previous exacerbation history may have caused the differences in FEV_1_ change.

In this study, we used blood eosinophil count as a marker of treatment response to ICS in COPD cohort. In consideration of patients with asthma-COPD overlap (ACO) high probability, we defined ACO by eosinophil count of cells/μL and/or extreme BDR criteria (> 15% and 400 mL). More than 98% were classified according to the eosinophil criteria. Absence of unified diagnostic criteria for ACO resulted in inconsistent clinical manifestations and outcomes. Moreover, in our previous study, we showed that blood eosinophil was the single most important biomarker to predict the decrease of exacerbation by ICS [28]. Recently, interests in precision medicine according to the subtype of COPD, rather than ACO is increasing. Blood eosinophil count is considered as the reliable biomarker in identifying subtype that might be benefitted by ICS. Although we did not find meaningful relation between FEV_1_ change and ICS use in eosinophilic COPD in real world data, additional prospective studies will be needed.

There were several limitations in this study. First, we analyzed subsequent 2 years of FEV_1_ data, which is a relatively short follow-up period. Second, we defined eosinophilic COPD using blood eosinophil counts at a stable period, and variation over time were not reflected. Third, COPD patients in our study were enrolled irrespective of treatment- naïve status. Whether blood eosinophil count less than or over 300cell/μL, the prescription rate for ICS containing inhalers was similar, which implies considerable numbers of subjects who have already been exposed to ICS are included, and this might be related to attenuation of ICS effects on eosinophilic COPD. Fourth, this was not a randomized clinical trial. Although the prescription rate for ICS containing inhalers was similar in both groups (41.1% and 40.7% in non-eosinophilic and eosinophilic groups, respectively.), ICS containing inhalers was not randomly assigned. ICS prescription was decided by pulmonologists. Thus, there can be a bias for the prescription of ICS. Caution is needed to interpret the impact of ICS on eosinophilic COPD. Fifth, changes in FEV_1_ according to GOLD classification could not be performed because approximately 90% of subjects were in GOLD group A or B. Lastly, we aimed to assess the rate of decline of FEV_1_ according to blood eosinophil count and whether FEV_1_ decline differs between eosinophilic COPD with and without ICS. Whittaker et al. [29] reported ICS use is associated with slower rates of FEV_1_ decline in COPD regardless of blood eosinophil level in a cohort of 26,675 COPD patients. On the other hand, Celli et al. [30] reported ICS plus LABA combination or the component, reduces the rate of decline of FEV_1_ in moderate to severe COPD. This study included more than 1,000 patients in each of subgroups. However, our study is underpowered to address intended questions about rate of decline of FEV_1_ due to small number of subjects.

## Conclusion

In conclusion, we found that the favorable longitudinal FEV_1_ change in eosinophilic COPD patients compared to non-eosinophilic COPD over 3 years in a Korean COPD cohort. ICS use did not have any beneficial effects on FEV_1_ change in eosinophilic COPD, but FEV_1_ decreased more rapidly with ICS use. Further studies with longer follow-up periods in larger number of patients are required.

## Supplementary Information

**Additional file 1.** **Supplementary Figure 1.** Values of FEV: over follow-up period in patients with eosinophillic and non-eosinophillic COPD.

## Data Availability

All data generated or analyzed during this study are included in this published article. Requests to obtain the raw data used for this study should be directed to the corresponding author, Chin Kook Rhee, at chinkook77@gmail.com.

## Abbreviations

AE: Acute exacerbation
ATS: American Thoracic Society
BDR: Bronchodilator response
BMI: Body mass index
CAT: Chronic Obstructive Pulmonary Disease Assessment Test
COPD: Chronic obstructive pulmonary disease
DLCO: Diffusion capacity of carbon monoxide
FEV_1_: Forced expiratory volume in 1 s
FVC: Forced vital capacity
GOLD: Global Initiative for Chronic Obstructive Lung Disease
ICS: Inhaled corticosteroid
LABA: Long acting beta2 receptor agonist
LAMA: Long acting muscarinic receptor agonist
mMRC: Modified Medical Research Council
PDE4 inhibitor: Phosphodiesterase-4 inhibitor
PY: Pack-year
SGRQ: St. George's Respiratory Disease Questionnaire
6MWD: 6-Min walk distance
