# Supplementary figures and images for: Longitudinal changes in forced expiratory volume in 1 s in patients with eosinophilic chronic obstructive pulmonary disease
Source: BMC Pulm Med. 2022 Mar 16;22:91. doi: 10.1186/s12890-022-01873-8 (PMC8925148; doi:10.1186/s12890-022-01873-8)

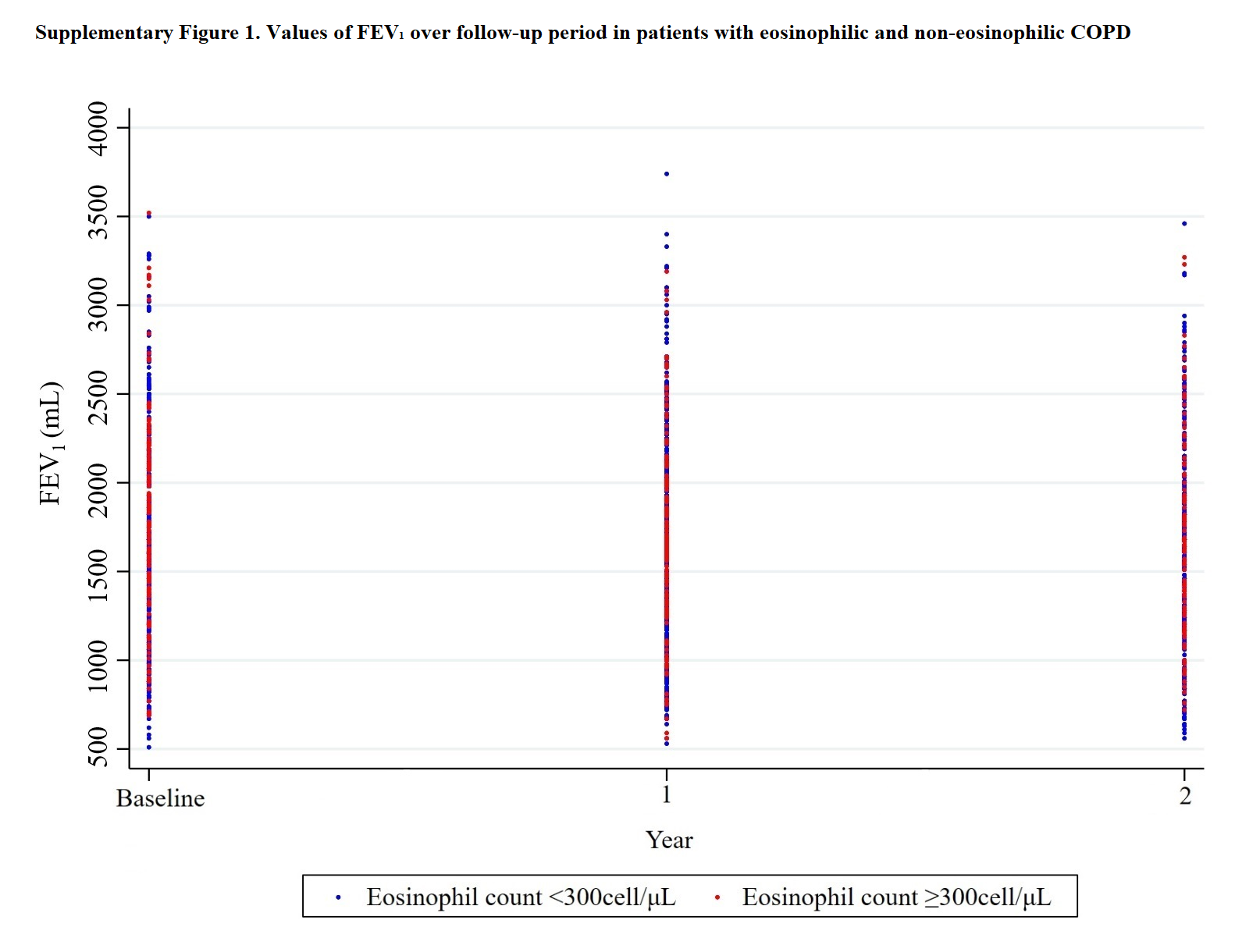

Supplement: Supplementary file 1 — Additional file 1. Supplementary Figure 1. Values of FEV: over follow-up period in patients with eosinophillic and non-eosinophillic COPD. [file 12890_2022_1873_MOESM1_ESM.tiff]
